# Origins of Chinese reindeer (*Rangifer tarandus*) based on mitochondrial DNA analyses

**DOI:** 10.1371/journal.pone.0225037

**Published:** 2019-11-13

**Authors:** Sheng-Nan Wang, Jian-Cheng Zhai, Wei-Shi Liu, Yan-Ling Xia, Lei Han, He-Ping Li

**Affiliations:** College of Wildlife Resources, Northeast Forestry University, Harbin, China; SOKENDAI (The Graduate University for Advanced Studies), JAPAN

## Abstract

The most southern population of reindeer (*Rangifer tarandus*) inhabits northeastern China, but the migration route and origin of this population have not been confirmed. The sequences of mitochondrial DNA control regions from domestic and wild herds from Eurasia and China were analysed. The results showed that the Chinese reindeer population originated independently from north-central Russian domestic herds, belonging to a large reindeer population that was present across Beringia during the last glacial period. Some studies have reported that the Chinese reindeer population is closely related to wild forest reindeer herds in Russia. Our results, however, indicate that wild forest reindeer herds of southeastern Russia contributed little or nothing to the Chinese reindeer herd gene pool. Chinese reindeer herds have a much greater genetic similarity to more northerly distributed tundra-type herds that inhabit open areas. The present findings will be essential for future conservation planning for Chinese reindeer.

## Introduction

The reindeer (*Rangifer tarandus*) is a keystone species in the Circumpolar North[1–3] and is widely distributed in Eurasian, North American and some Arctic islands. As this species survived the last glacial period, studies on reindeer fossils and migratory routes have provided reliable materials for phylogeographical research [4–8]. Man-made antler punches found in Hunker Creek and at the Udora site in southern Ontario that are approximately 11300–10550 years old are considered the earliest signs of interaction between reindeer and humans [9]. Hedman concluded that herding and husbandry of reindeer probably developed beginning in the last part of the Iron Age [10]. In the past thousands of years, reindeer have constituted an important biological resource for the physical and cultural survival of Arctic residents and have been exploited for food and other necessary subsistence materials[11, 12]. The species was probably essential for human immigration and colonization of the Eurasian Arctic and Subarctic [13, 14]. At the same time, domestic reindeer have provided great contributions to the origin and development of many northern indigenous cultures [11, 15–17]. There are two competing hypotheses addressing the origin of reindeer herding. One hypothesis suggests that domestic reindeer first appeared a few thousand years ago east of the Urals in the southern part of the Siberian taiga, from where they further spread to other regions. On the other hand, the other hypothesis suggests that the domestication of reindeer occurred independently multiple times in different parts of Eurasia [14, 18, 19]. Similarly, researchers have disputed the origin of reindeer. Recently, genetic studies have demonstrated that mitochondrial DNA (mtDNA) is a useful marker for research on past origins and evolutionary time scales [20]. Based on mtDNA, different refugia and colonization routes have been suggested for a range of roaming terrestrial Arctic species such as the collared lemming (*Dicrostonyx groenlandicus*)[21], the reindeer (*R*. *tarandus*) [22], the Arctic fox (*Alopex lagopus*)[23] and the wolverine (*Gulo gulo*)[24]. Kvie found that some reindeer colonized Arctic islands from the Eurasian mainland through natural dispersal based on mtDNA analysis [3]. Røed reported the origin of reindeer herding and the domestication history of domestic reindeer and wild caribou throughout Eurasia based on mtDNA analysis [25].

At present, the southernmost reindeer populations of the world are distributed in Genhe, north of the Greater Khingan Mountains in Inner Mongolia, China [26–28]. The Chinese reindeer population was semi-domesticated by the Ewenki people and lost its migratory habits. Now, the reindeer along the northern Greater Khingan Mountains are distributed in 8 small, stable populations. In this habitat, mountains and traffic restrict the movement of the reindeer between locations, and mixing phenomena rarely occurs, limiting gene exchange. Some early Chinese studies speculated that reindeer herds were introduced to China from the Lena River Basin in Western Siberia and the Yakutia in the Lake Baikal basin of Russia by Ewenki ancestorrs in the eighteenth century and that they belong to a Siberian woodland reindeer subspecies (*R*. *tarandus valintinae*)[27]. However, there is not ample evidence to clarify the migration route and origin of Chinese reindeer, especially at the genetic level. Therefore, we sought to obtain the following information by analysing the D-loop and samples from Russia, Norway, and Finland: 1) the genetic structure of Chinese reindeer; 2) the relationship of this reindeer population with Eurasian reindeer herds; and 3) the real origin of this population.

## Materials and methods

### Samples

**Fig 1:**
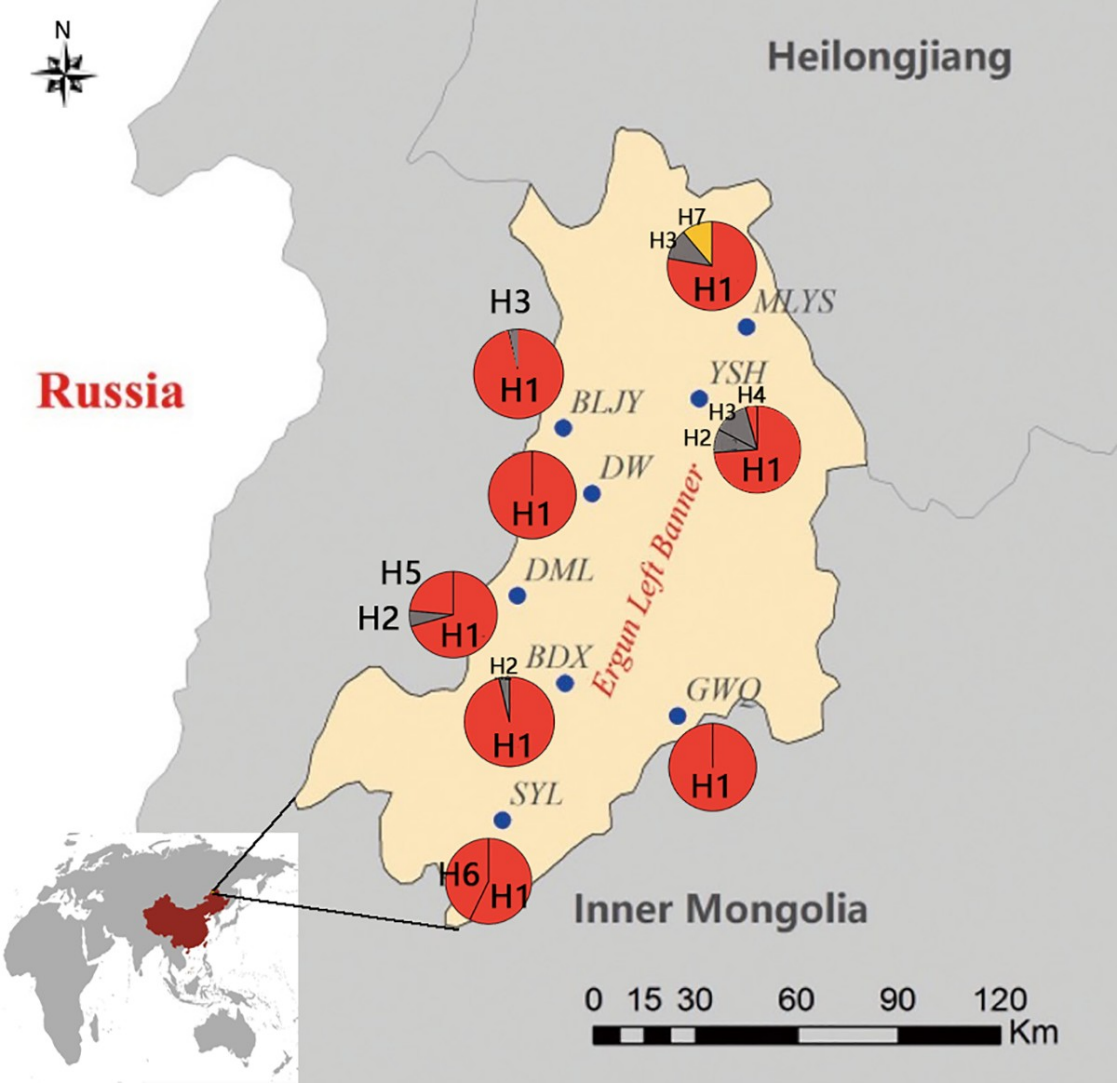
Distribution of *Rangifer tarandus* sampled from eight geographical regions in China. The eight reindeer clusters are detailed in Table 1,and the haplotypes in the pie chart are colored according to the haplogroup classification in Fig 3A. Pale-yellow are on the map indicates the distribution range of Chinese reindeer, which can be found in Fig 3B.

Faecal samples were collected across the range in the winter seasons of 2013–2014 with the help of the local Ewenki within a few days of a snowfall. The reindeer were labelled with colour ribbons tied on their necks or were identified by other characteristics, such as body size, to avoid repeat sampling from the same individual. This marking of the reindeer was approved by the Ewenki peoples who coexisted with the reindeer. The Ewenki people helped control the reindeer by calling them reindeer and giving salt rewards without anaesthesia. The labels were removed in the same manner at the end of the experiment. No animals were harmed in this experiment. The experiment complied with animal ethics guidelines. Following their collection, the faecal samples were bagged, frozen and shipped to a laboratory for analysis. DNA was extracted from the tissue present in the outer mucosal layer of each sample. The geographical distribution of the samples is shown in Fig 1, and the geographical positions are listed in Table 1. A total of 426 mtDNA samples from Finland, Norway and Russia (central, northwestern, southeastern, and northeastern) were obtained based on the previous study [25]. We adhered to the legal requirements of China in our research.

**Table 1:** Information on the faecal samples of the reindeer.

Name and abbreviation of location	No. of samples	Longitude and latitude	Sampling year	No. per haplotypes
Yang Shuanghu hunting sites(YSH)	24	N51°41.490', E122°15.257'	2013	H1-17; H2-3; H3-3; H4-1
Budongxia hunting sites(BDX)	27	N51°00.273', E121°24.609'	2013	H1-26; H2-1
Balajieyi hunting sites(BLJY)	28	N51°41.593', E121°38.217'	2013	H1-25; H3-1
Damala hunting sites(DML)	21	N51°16.026', E121°17.135'	2013	H1-12; H2-1; H5-4
Dawa hunting sites(DW)	27	N51°30.008', E121°42.105'	2014	H1-27
Guwenqiang hunting sites(GWQ)	20	N50°51.089', E121°51.612'	2013	H1-20
Suoyuian hunting sites(SYL)	7	N50°40.217', E121°01.387'	2014	H1-4; H6-3
Maliyasuo hunting sites(MLYS)	9	N51°51.358', E122°31.612'	2013	H1-7; H3-1; H7-1
Total	163			

Number of haplotypes (“-number”), haplotypes(H)

### DNA extraction

Total genomic DNA was extracted from the mucosal coats of the faecal pellets using a QIAamp DNA Stool Mini Kit (Qiagen, Germany) following the manufacturer’s instructions and with some optimizations. A UV-visible light spectrophotometer (NanoDrop 2000c, Thermo Scientific, USA) was used to detect the content and purity of the extracted DNA. The extracts were frozen at -20°C [29].

### PCR and sequencing

A 429 bp mtDNA control region fragment was amplified and sequenced from a subset of 153 Chinese reindeer (GenBank accession numbers MG604581-MG604733) using the L15394 and H15947, primers provided by previous study [22] (L15394: 5’- AAT AGC CCC ACT ATC AGC ACC C-3’ and H15947: 5’- TAT GGC CCT GAA GTA AGA ACC AG-3’). Polymerase chain was performed on a total volume of 20 μl/sample containing 10 ng of DNA, 1× PCR buffer, 1.5 mM MgCl_2_, 0.2 mM of each dNTP, 2 μM of each primer, 0.4 units of *Taq* polymerase (Invitrogen), and 0.1 mM BSA. The PCR conditions were as follows: an initial denaturation step of 5 min at 94°C followed by 30 amplification cycles. Each amplification cycle consisted of denaturation for 30 s at 94°C, annealing for 30 s at 56°C, and extension for 30 s at 72°C. A final extension time of 2 min at 72°C was added after completion of the amplification cycles. All samples were visualized with ethidium bromide in a 1.5% agarose gel electrophoresis for 60 min at 120 volts. For successfully amplified samples, the PCR products were purified using a master mix of 0.90 μl of Antarctic phosphatase buffer, 0.10 μl of Antarctic phosphatase, and 0.03 μl of exonuclease I (New England BioLabs) per 7 μl of each PCR product. The purified prouducts were at 37°C for 15 min and then subjected to a second incubation step at 80°C for 15 min. For cycle sequencing reactions (12 μl/sample), 1.0 μl of 5× sequencing buffer, 0.5 μl of BigDye Terminator mix v3.1 (Applied Biosystems), 0.8 μM of primer, and 0.5 μl of purified PCR product was used. The cycle sequencing conditions were as follows: an initial denaturation step at 96°C for 2 min was followed by 30 amplification cycles consisting of a denaturation step at 96°C for 20 s, an annealing step at 50°C for 20 s, and an extension step at 60°C for 4 min. The DNA sequencing was pergormed by Jilin Comate Biosciences Co., Ltd., Changchun City.

### Quality checks and alignment

All sequence data were thoroughly checked visually and edited manually. In addition to the generated sequence data from this study, control region sequences from several previous studies [22, 25, 30], were downloaded from GenBank and compiled in a single FASTA file. A few sites with ambiguous sequences were discarded to ensure the high-quality alignment and to confidently identify haplotypes. All of sequences were trimmed and aligned (using Clustal X implemented in MEGA) [31] using the program MEGA 5 [32] and were checked visually afterwards. The program DNaSP v5 [33] was used to identify control region haplotypes and to calculate the polymorphic site numbers and nucleotide diversity (Table 2).

**Table 2:** Geographical origin, status and polymorphism in the sampled reindeer populations.

Geographical origin	N	H	Hd	π
China	153	7	1.000	0.01367
Russia domestic	105	35	0.997	0.01539
Russia wild	30	15	1.000	0.01413
Norway domestic	73	11	1.000	0.01618
Norway wild	138	29	0.995	0.01699
Finland	56	9	1.000	0.01701
Total	555	106	-	-

Sample size (N), number of haplotypes (H), haplotype diversity (Hd) and nucleotide diversity (π).

### Phylogenetic analysis

To prevent additional outgroup species from introducing a high number of gaps, the moose (Alces alces, GenBank accession number U12866) was chosen as an outgroup to ensure consistency with the study by [30, 34]. Sites, including gaps, were used in this study. The best model (GTR+I+G) was selected by Akaike’s information criterion(AIC) in Modeltest 3.7 [35]. Bayesian inference (BI) was performed with MrBayes 3.2.6, and four Markov chains were electrophoresis for five million generations, with sampling every 100th generation. The first 25% of the trees were discarded [36].

### Network analysis

Phylogenetic methods may not lead to the desired resolution at the intraspecific level due to the lower genetic diversity and non-hierarchical nature of intraspecific data sets compared to interspecific data sets [35, 37]; complementary network approaches might be valuable alternatives for studying phylogenetic structures and haplogroups at the population level. A network approach might be most appropriate in the case of reindeer because some of the subspecies/ecotypes are known to be migratory [38], and relationships may be better captured by a network than by a bifurcating tree in this case. Therefore, the program NETWORK 4.6.1.0 [39] was used to reconstruct median-joining trees and networks.

## Results

### Summary statistics

**Fig 2:**
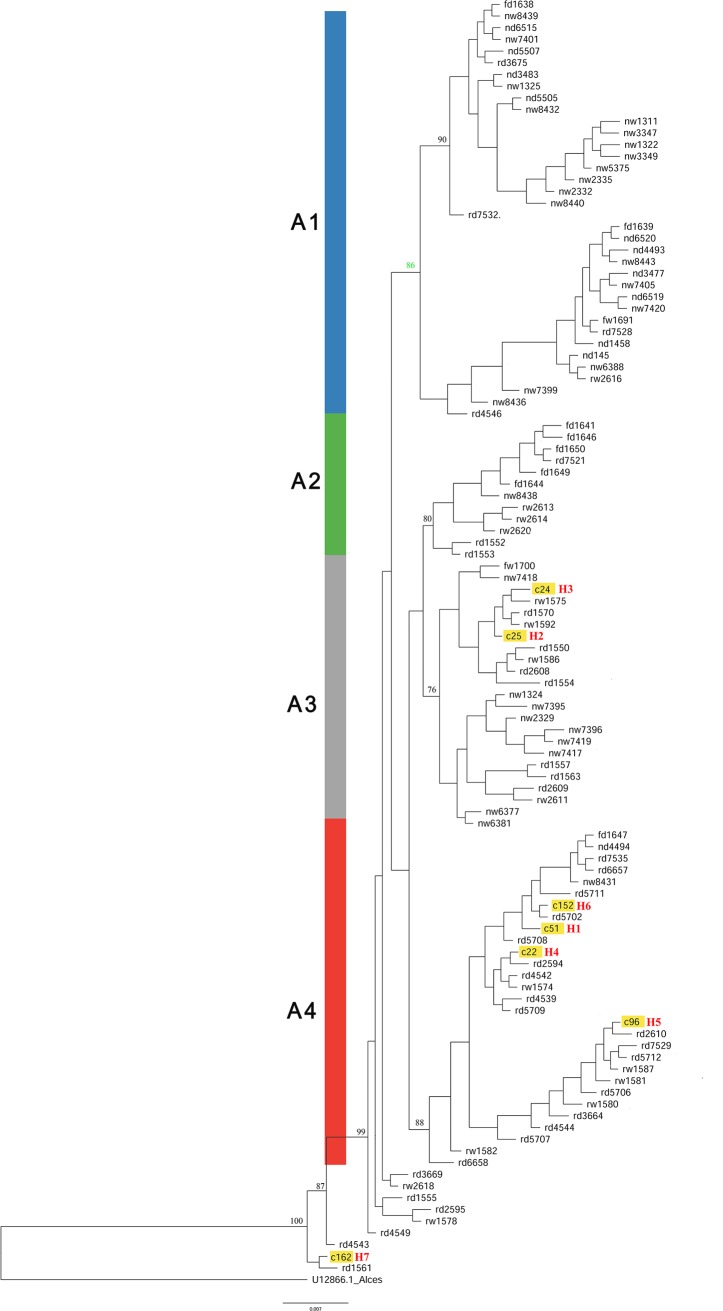
Bayesian phylogenetic tree reconstruction. **Bayesian phylogenetic tree reconstruction using mitochondrial control region haplotypes.** The coloured branches represent the haplogroups (blue = A1, green = A2, grey = A3, red = A4). The haplotypes marked in red were found only in Chinese reindeer populations. Node values = BPP×100%, only node values≥0.75 are indicated.

The data included six populations in Russia, two populations in Norway, two populations in Finland and eight smaller clusters in China. Eighty-one different mitochondrial haplotypes were identified in 579 reindeer samples. For Chinese reindeer, 7 haplotypes could be identified among 153 samples, of which 3 haplotypes are reported for the first time. Further information regarding the spatial distribution of the mitochondrial haplotypes, including frequencies, can be found in Table 1 and Fig 2.

### Phylogenetic and network analysis

**Fig 3:**
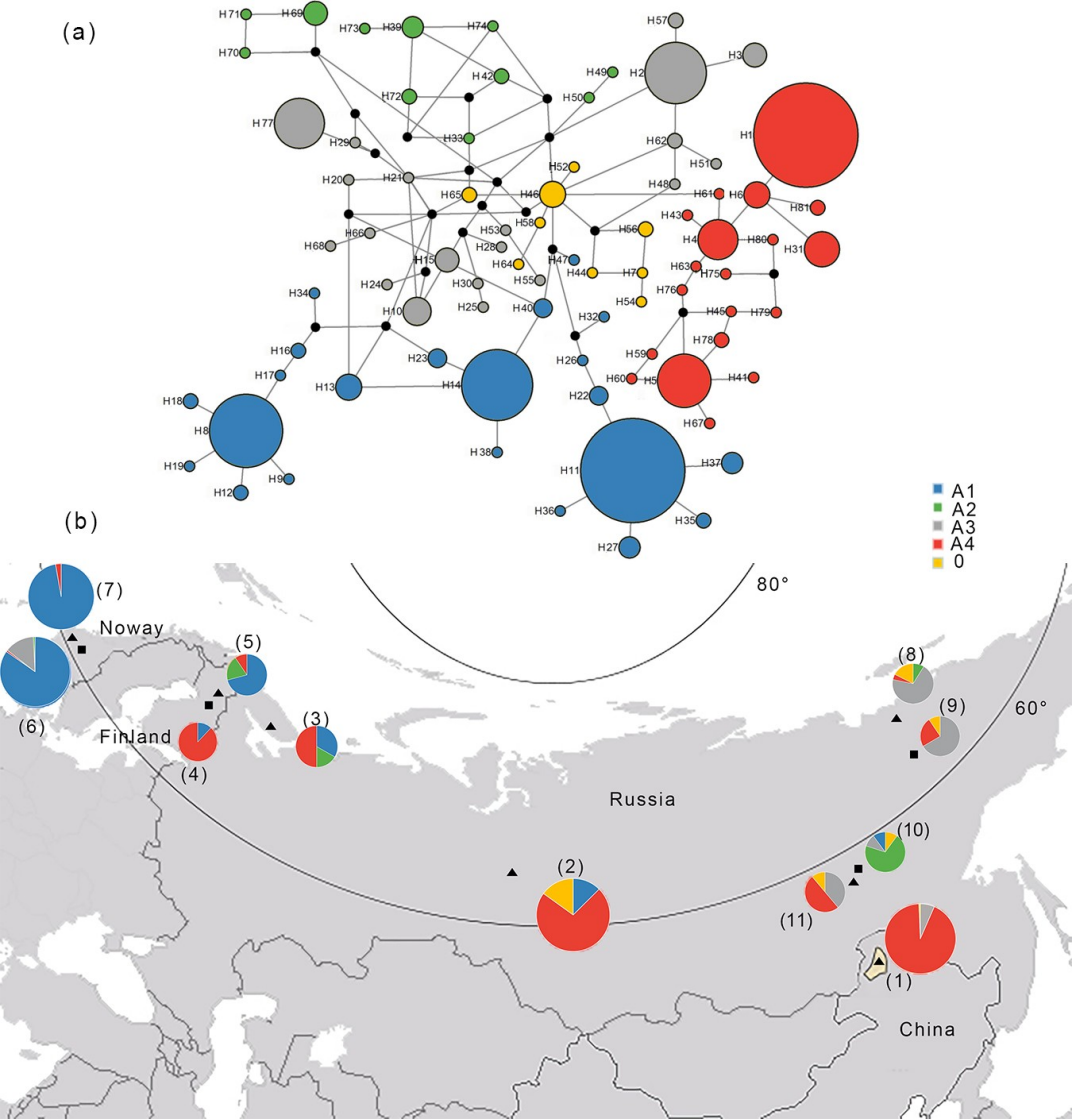
**(a) Median-joining network for mitochondrial DNA haplotypes found in Chinese reindeer.** The five different haplotype clusters (haplogroups, A1-A4 & 0) are encircled with different colours. The circles represent haplotypes, and the circle sizes are proportional to the haplotype frequencies. **(b) Haplogroup frequencies in wild (squares) and domestic (triangles) reindeer herds.** The populations were pooled according to geography and whether they were wild or domestic herds. (1) Northeastern China; (2) north-central Russian domestic; (3) northwestern Russian domestic; (4) Finland wild; (5) Finland domestic; (6) Norway wild; (7) Norway domestic; (8) northeastern Russian domestic; (9) northeastern Russian wild; (10) southeastern Russian wild; (11) southeastern Russian domestic.

Zhai’s studies based on microsatellite markers to demonstrated that Chinese reindeer are divided into eight smaller populations, and our study with this conclusion based on analysis of mitochondrial DNA[40]. The median-joining network (Fig 3A) revealed an internal assemblage of haplotypes connected to sampling location (Fig 1), helping to demonstrated the relationship between these factors (Haplotypes, Phylogenetic, Genetic, Evolutionary distance, Sample area). The distribution of different haplotypes in China can be observed in Fig 1. The H1 haplotype had a very high proportion and comprised the Dawa (DW) and Guwenqing (GWQ) clusters. Seven haplotypes of the Chinese reindeer population were identified in five haplotype clusters shown in the network. As shown in Fig 3A, haplotypes H1, H4, H5, and H6 (red) were found in the DW, GWQ hunting sites; H2 and H3(grey) were found in the Budongxia (BDX), Damala (DML), Balajieyi (BLJY), Yang Shuanghu (YSH) and Maliyasuo (MLYS) hunting sites; and H7 (yellow) was found only in the MLYS hunting sites. Compared to the Eurasian reindeer populations, the Chinese population had medium haplotype diversity and the lowest nucleotide diversity (Hd 1.000, 0.01367)(Table 2).

**Fig 4:**
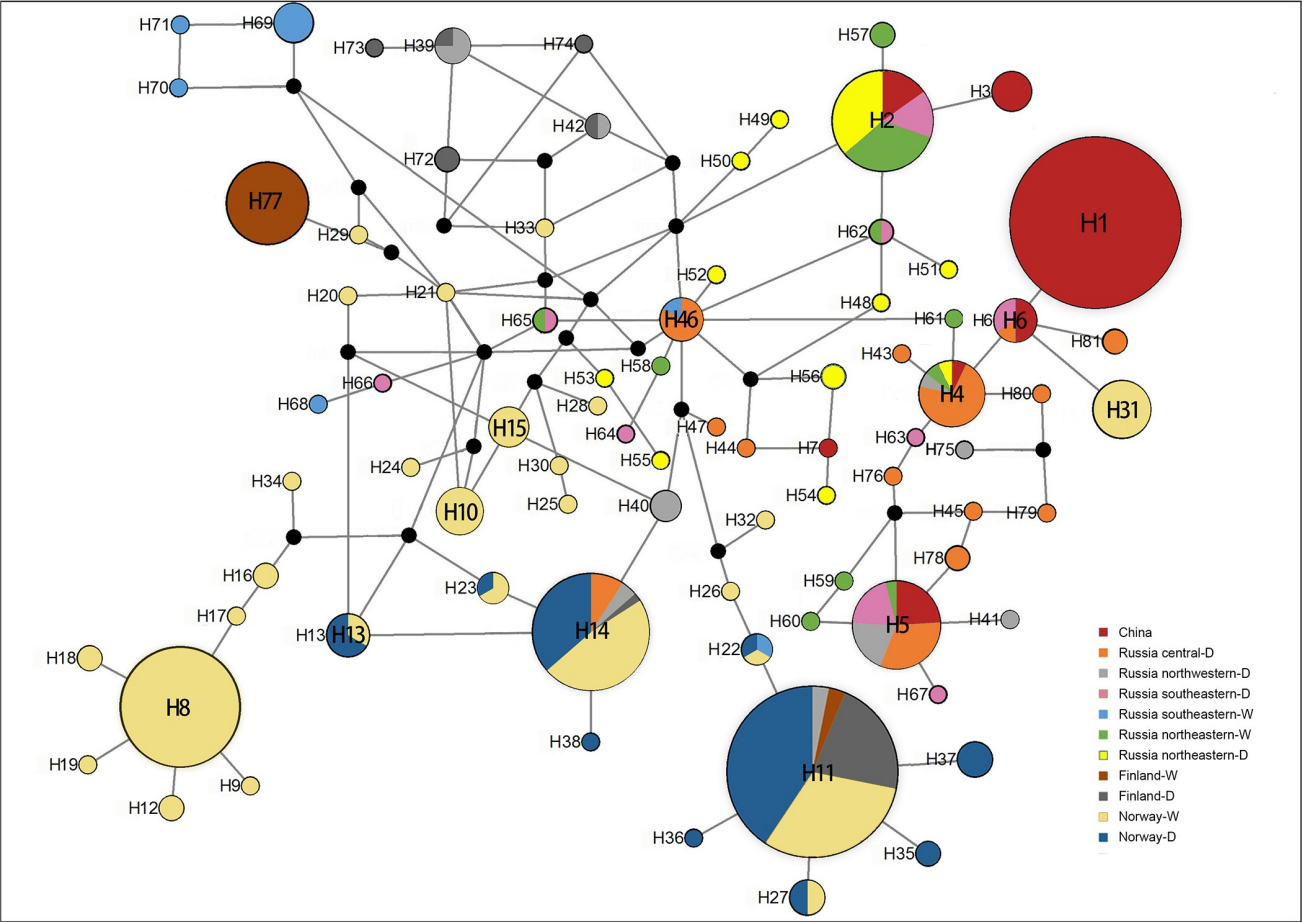
Median-joining tree. **Median-joining tree of the mtDNA control region haplotypes identified in this study.** The circles represent haplotypes, and the circle sizes are proportional to haplotype frequencies. The circles are coloured according to the haplogroup membership locations.

Our research showed that haplotypes H1, H3, and H7 were found only in the Chinese reindeer population (Fig 4). DW, GWQ hunting sites were composed only of haplotype H1, which was the largest haplotype in the Chinese reindeer population. As shown in the tree, H1 in Eurasian populations of central Russia and the high Arctic region had the same lineage with high support. Fig 4 shows that H2, which existed in the DML and YSL Chinese hunting sites, was also found in reindeer populations in eastern Eurasia. H3 was found at the YSL, MLYS and BLJY hunting sites. In our study, the H4 and H5 haplotypes were rarely found in the Chinese population but were commonly found in central Russian herds.

In the Bayesian phylogenetic tree and network, four highly supported lineages (A1-A4) could be identified. The Eurasian reindeer, including the Norwegian herds, the Finnish herds, the different Russian herds and the Chinese herds, were divided into four major haplogroups (Fig 2, A1, A2, A3, and A4) in the Bayesian phylogenetic tree, which corresponded to the median-joining network (Fig 3A). Haplogroups A1-A2 haplogroup consisted of 33 reindeer samples haplotypes (247 individuals). Haplogroup A3 consisted of 21 haplotypes (67 individuals). Haplogroup A4 was consisted of 19 haplotypes (228 individuals). North-central Russian domestic reindeer were distributed in A1 and A4, and 73% of them fell into the A4 group. Most of the northeastern wild reindeer fell into the A3 (67%) and A4 (24%) groups. A total of 99.3% of the Chinese samples fell into the A3 and A4 haplogroups and were very close to the north-central Russian domestic reindeer and northeastern wild reindeer. According to the map of haplogroup membership (Fig 3B), a gradual separation of lineages became apparent, with the A1 haplotypes mainly distributed in northern Norway and Finland, whereas haplogroups A1 and A3 had overlapping distribution ranges in northwestern Russia. In other words, the A3 haplotypes had high proportions in the northeastern Russia, whereas the A3 and A4 haplotypes had overlapping distribution ranges in southeastern Russia and northeastern China. Notably, A2 haplotypes were found only in a few individuals of eastern Finland and southeastern Russia (wild). Nineteen haplotypes were found in haplogroup A4; among them, only 16(80%) were found to be present in large proportions in Russian domestic reindeer. The A2 haplogroup was divided into two smaller independent clusters; one cluster consisted of Finnish domestic herds, and the other consisted of southeastern Russian wild herds (Fig 4).

Additionally, we plotted the geographical distributions of the haplotypes for each haplogroup (Figs 3 and 4). This analysis revealed that the majority of individuals carrying A4 haplotypes were found in central of Eurasian (including northeastern of China), whereas the majority of individuals carrying A3 haplotypes were found in eastern of Eurasia.

## Discussion

Our analysis clearly identified that the H1 haplotype was the most common and dominant in all Chinese reindeer populations. The genetic similarity between these Chinese reindeer populations indicates gene flow and dispersal from a common source population and supports the theory that these hunting sites were likely colonized from the Eurasian mainland. The Chinese reindeer herds began to inhabit in the Greater Khingan Mountains approximately 300 years ago and have remained in physiogeographical isolation; gene exchange between populations is almost impossible. The H1 haplotype from cluster A4 was found only in Chinese reindeer and was present in a high proportion (86%). However, the presence of the H1 haplotype in the Chinese populations could be the result of random haplotype survival from a common refugial population. More extensive sampling of Eurasian wild and domestic reindeer, with a special emphasis on southeastern Russian reindeer, would be necessary to clarify this possibility further. The southeastern Russian population was found to be the origin of Chinese reindeer in the previous studies. However, contrary to previous studies, we found that the H1 haplotype was included in the genetic A4 cluster of the domesticated reindeer in central Russia. Our data support the hypothesis that Chinese reindeer were most likely originated from north-central Russian domestic herds, not from woodland reindeer, as previous studies have suggested[27]. Haplotype sharing was very limited between southeastern Russia and Chinese reindeer (Fig 3B), in contrast to findings from previous studies. In the median-joining network, the star-like cluster H1 was connected to the central haplogroup 0 (Fig 3A). Haplogroup 0 (Fig 3A) was present in eastern Russian and Chinese populations. Indirectly, not only H1 but also all haplotypes are connected to haplogroup 0. Among the haplotypes, H46 was found in the central of the network, and has experienced obvious expansion. This implies that Chinese reindeer herds and northeastern Eurasian reindeer populations have the same ancestral haplotype. The phylogenetic tree was reconstructed based on haplotype, separating wild woodland reindeer from another domestic reindeer subspecies, consistent with studies by Klütsch [22, 30, 41]. H5 was occupied by a relatively small proportion of Chinese reindeer populations and was observed in another cluster of the A4 lineage, close to southeastern domestic Russian reindeer herds. This finding suggests that the gene pools in the southeastern Russian and Chinese populations have met and mixed. This distribution supports two scenarios. First, these herds may have come from the same geographically well-separated refugial origins. Second, reindeer from these herds may have been exchanged by the reindeer herding peoples of central and southeastern Russia and China.

Thus, the north-central Russian and Chinese reindeer, which belonged to the large haplotype assemblages A3 and A4, may have originated from Beringia during the last glacial period. The spatial distribution of haplotype lineages A1-A4 (Fig 3) supported the scenario of two geographically well-separated refugial origins. If only one glacial refugium or two refugia existed in one geographically restricted area, one would expect that the lineages might have mixed early in the expansion and subsequently spread throughout Eurasia with relatively similar haplotype proportions. This was clearly the case, since there was a gradual separation in the Eurasian herds from the west to central areas, although there were overlapping zones between the Finnish and northwestern Russian herds, sharing of the other line between the eastern Eurasian herds and the southern Chinese herds was supported by the number of mtDNA haplotypes (Fig 3). Parts of the Russian wild/domestic reindeer herds, which come from different geographical regions, shared the same haplotype with Chinese reindeer herds in the haplogroups A3, A4 and 0. Haplogroup 0 (Fig 3A) was represented by both eastern Russian and Chinese reindeer. Among the haplotypes, H46 was found in the centre of the median-joining network and had experienced obvious expansion. This finding shows that Chinese herds are slightly different from the herds from those areas and are obviously different from Fennoscandian and southeastern Russian wild herds. The wild populations in Finland and central Norway have contributed little or nothing to the Chinese reindeer herd gene pool. In addition, the wild reindeer residing in the mountain taiga in southeastern Russia showed a genetic composition that was markedly different from that of the local domestic herds. Interestingly, Chinese reindeer with haplotypes H2, H3, and H7 had close relationships with northeastern Russian reindeer populations (Fig 4). In fact, the Chinese reindeer herds had a much greater genetic similarity to the more northeastern Russian wild and northeastern Russian domestic herds than to other herds (Fig 3B). Therefore, the Chinese reindeer used in the domestication process probably derived from large tundra herds instead of smaller herds residing in the forest. The tundra type that inhabits open areas is more gregarious than the forest-dwelling types; in addition, it has evolved a more sophisticated social organization[42], and is more easily domesticated than the smaller herds in the Eurasian forests. From ecological and physiogeographical perspectives, the reindeer living in the Greater Khingan Mountains of northeastern China, which form the southernmost reindeer population in the world, probably adapted to the climate in the Greater Khingan Mountains to avoid predatory pressure and intraspecific competition in the lowlands. The same phenomenon was also found in a study by Guthrie and Matthews [43]. The expanding forest may have driven the reindeer populations to migrate further south where the warmer climate facilitates the growth of various vascular plants and mushrooms, thus expanding the food base for the reindeer in China. However, isolation of habitat has led to haplotype diversity in the Chinese reindeer herds at low levels. In fact, habitat fragmentation, extensive management, pervasive inbreeding, and no or limited exchange between populations outside of Chinese reindeer herds could be partly or entirely responsible for this situation. Therefore, these reindeer are at particular risk. As discussed previously, we suggest importing reindeer from the large tundra herds of the northeastern Russia to increase the genetic diversity of the Chinese reindeer herds and to improve the clustering structures of these herds. This situation necessitates proper management plans with an emphasis on conserving genetic variability in Chinese reindeer. This study has revealed strong genetic similarities found between Chinese and Russian reindeer populations. The study lends considerable weight to the hypothesis that these Chinese reindeer populations may have colonized via a route from the Eurasian mainland. Moreover, this study strongly disproves the hypothesis that these populations of Chinese reindeer are feral populations of introduced Siberian woodland reindeer (*R*. *tarandus valintinae*)[27]. It is our hope that these important results will help clarify the origin of the Chinese reindeer that from the southernmost reindeer population and aid in conserving this important and unique population.
